# Mechanically Rotating Intravascular Ultrasound (IVUS) Transducer: A Review

**DOI:** 10.3390/s21113907

**Published:** 2021-06-05

**Authors:** Jin-Ho Sung, Jin-Ho Chang

**Affiliations:** Department of Information and Communication Engineering, Deagu Gyeongbuk Institute of Science and Technology, Daegu 42988, Korea; madeinjinho@dgist.ac.kr

**Keywords:** intravascular ultrasound (IVUS), atherosclerosis, mechanical rotating IVUS transducer, single frequency, multifrequency

## Abstract

Intravascular ultrasound (IVUS) is a valuable imaging modality for the diagnosis of atherosclerosis. It provides useful clinical information, such as lumen size, vessel wall thickness, and plaque composition, by providing a cross-sectional vascular image. For several decades, IVUS has made remarkable progress in improving the accuracy of diagnosing cardiovascular disease that remains the leading cause of death globally. As the quality of IVUS images mainly depends on the performance of the IVUS transducer, various IVUS transducers have been developed. Therefore, in this review, recently developed mechanically rotating IVUS transducers, especially ones exploiting piezoelectric ceramics or single crystals, are discussed. In addition, this review addresses the history and technical challenges in the development of IVUS transducers and the prospects of next-generation IVUS transducers.

## 1. Introduction

### 1.1. History of IVUS Transducers

The development of intravascular ultrasound (IVUS) transducers began with efforts to monitor the heart. After Edler and Hertz [1] first demonstrated that ultrasound can be used to monitor the actual movement of the heart in 1954, studies on catheter-based intracardiac ultrasound transducers were vigorously carried out to measure the structure and movement of the heart and vessels. In the 1960s, Cieszynski [2] reported a miniaturized ultrasound transducer inserted into the heart lumen through the jugular vein of a dog, and Kossoff [3] developed an 8-MHz ultrasound transducer with a 2-mm diameter, which was mounted on an 8F Cournand catheter. Carleton et al. [4] presented a 2.25-MHz nondirectional cylindrical element transducer mounted on an 8F catheter, and two element transducers mounted on a catheter were suggested by Peronneau [5], Stegall et al. [6], and Kardon et al. [7]. In addition, many IVUS transducers using Doppler signals have been investigated to measure blood flow [8,9,10,11]. However, although these transducers were inserted into the vessel like the current IVUS transducer, they were unable to provide a cross-sectional image of the vessel. 

In 1955, Wild [12] developed a single-element ultrasound endoscope, which is a mechanically rotating transducer for the detection of rectal tumors. Starting with this, ultrasound transducers, most similar to the current single-element IVUS transducer, began to be reported. Various IVUS transducers rotating and being pulled back were introduced [13,14,15] to visualize cross-sectional images of the heart and vessels by C-scan, and Wells [16] employed a rotating mirror to obtain a vascular cross-sectional image. In 1972, a solid-state IVUS transducer, a phased array transducer type, was developed by Bom et al. [17]. It can obtain cross-sectional images without mechanical movement. Thereafter, IVUS transducers were developed using these two approaches, the mechanical IVUS transducer and the solid-state IVUS transducer, which are discussed in detail in Section 4, and their role has focused on diagnosing atherosclerosis as well as monitoring stent surgery.

### 1.2. Atherosclerosis

Atherosclerosis is a cardiovascular disease that causes an ischemic stroke or acute coronary syndrome. By building plaque inside the vascular wall, it narrows down the arterial lumen area and leads to stenosis or thrombus [18,19,20,21,22]. Because it develops gradually over years, it often is not accompanied by notable symptoms until it becomes severe [18,23,24]. 

**Figure 1:**
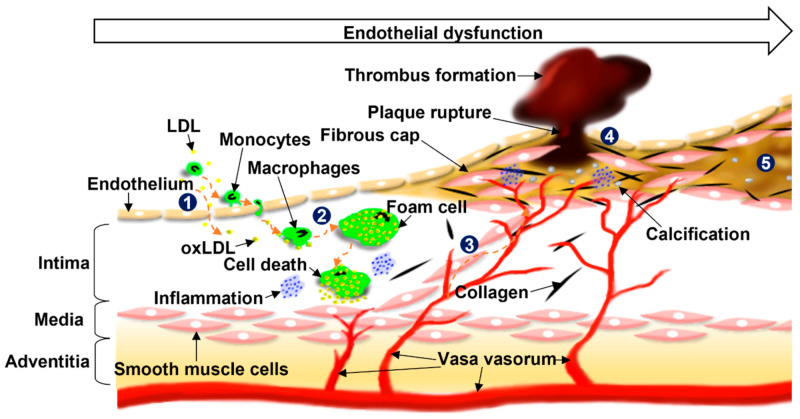
Development of atherosclerosis. (1) Dysfunction of endothelial cells (ECs) leads to migration of low-density lipoproteins (LDLs), resulting in the expression of monocyte adhesive molecules on the ECs and inflammation. (2) Monocytes transmigrate intima and differentiate to macrophages binding to oxidized LDL (oxLDL), which causes the formation of foam cells and cell death. Fatty streaks are developed and vasa vasorum begins to be built. An additional increase in foam cells and macrophage necrosis induces core of extracellular lipid and calcification of the plaque occurs. (3) Successive inflammation gives rise to the migration of smooth muscle cells with collagens from the media to the intima forming a fibrous cap. (4) Further accumulation of plaque and inflammation causes the thickness of the fibrous cap to be thin, and eventually, the plaque’s fibrous cap ruptures, forming thrombus. (5) Plaque stabilizes, resulting in a stenosed lumen area [25,26].

Figure 1 illustrates the development of atherosclerosis [25,26]. The normal artery is composed of three layers: the intima, media, and adventitia. The intima has a single layer of endothelial cells (ECs) in contact with the blood flow, and ECs control vascular functions, such as vascular tone, thrombogenicity, and inflammation [27,28,29,30]. When ECs are activated by injury or dysfunction, they increase the permeability of low-density lipoprotein (LDL) particles, making the migration of LDL particles into the subendothelial space possible. In the space, the particles are modified to oxidized LDL (oxLDL) and lead to the expression of monocyte (or leukocyte) adhesive molecules on the ECs, which drives inflammation. Through the adhesive molecules, monocytes transmigrate into the intima and then differentiate into macrophages, which engulf oxLDL, resulting in foam cells [31,32]. Fatty streaks that contain lipid-filled macrophages develop, and vasa vasorum (VV) begins to be built for the local vascular supply [18,22].

With the increase in foam cells and macrophage necrosis in the subendothelial space, small extracellular lipid pools develop, and, eventually, a core of extracellular lipid forms. Meanwhile, further inflammation occurs, and, subsequently, smooth muscle cells and collagens migrate from the media to the intima, resulting in the formation of a fibrous cap over the lipid pool. Calcification of the plaque begins with these types of lesions. The additional accumulation of plaque and inflammation causes the fibrous cap to be thin; then, the plaque can rupture easily. This plaque is described as a “vulnerable plaque,” which means it is unstable [33,34,35,36]. 

When the plaque ruptures, coagulation factors in the blood are activated, forming a thrombus. If the thrombus is large enough to obstruct blood flow or is stuck in some small vessels by straying from the original lesion location, acute cardiovascular events appear. In addition, even though the ruptured plaque is healed successfully, the thickened intima is more likely to cause severe stenosis [18,21]. Thus, it is extremely important to assess plaque vulnerability in the early stages of the disease because approximately 70% of acute cardiovascular events are caused by plaque rupture [23,24]. As a result, the goal of IVUS imaging is to determine the morphological changes caused by atherosclerosis for the diagnosis of cardiovascular diseases and to determine the treatment direction.

## 2. Diagnostic Indicators for Atherosclerosis

In this section, several indicators in the diagnosis of atherosclerosis are based on IVUS images. Even though there is controversy over the indicators because various factors are involved in the development of atherosclerosis, looking into current diagnostic indicators can help understand the future direction of IVUS transducer development.

### 2.1. Plaque Burden

**Figure 2:**
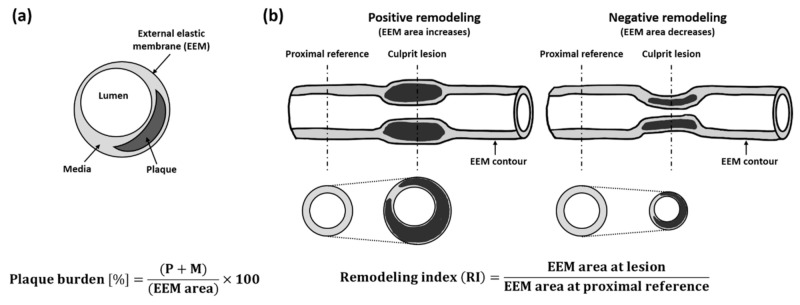
Measurements of (**a**) plaque burden and (**b**) remodeling index (RI). P and M are areas of plaque and media, respectively, and EEM indicates the external elastic membrane. P + M is also equal to EEM area–lumen area [37,51].

The plaque burden denotes the amount of plaque in the vessel wall, and it is generally calculated using the following Equation (1) [37,38,39].

(1) $$\mathrm{Plaque}\;\mathrm{burden}\;\left[\%\right]=\frac{\left(P+\;M\right)}{\left(\mathrm{EEM}\;\mathrm{area}\right)}\times 100$$

where P and M indicate areas of plaque and media, respectively. The external elastic membrane (EEM) is located at the outer boundary of the media, as shown in Figure 2a. The P + M area corresponds to the difference between the EEM and lumen areas. Because plaque formation is a representative symptom of atherosclerosis, there have been many efforts to predict the risk of disease by plaque burden [40,41,42,43,44,45,46]. It is known that a larger plaque burden (>70%) with a minimum lumen area (<4 mm^2^) in non-culprit lesions can be a criterion to predict future cardiovascular events [42,43,44]. For the accurate measurement of plaque burden, it is essential to clearly identify the boundaries of each blood vessel layer. This requires the high spatial and contrast resolutions of IVUS imaging, as well as an imaging depth deeper than 3 mm. 

### 2.2. Arterial Remodeling Index

Although arterial remodeling primarily refers to any changes in the vascular wall caused by aging, injury, or disease, in the field of vascular disease, it mainly indicates a change in vessel size [47,48]. When diseases occur, blood vessels begin either to expand (i.e., positive remodeling) or constrict (i.e., negative remodeling) as a reaction to pathophysiologic conditions. The extent and type of arterial remodeling can be represented by the remodeling index (RI), which is the ratio of EEM in the culprit area to the EEM area in reference, as shown in Figure 2b. For example, RI >1.05 indicates positive remodeling, and RI <0.95 denotes negative remodeling. An RI between 0.95 and 1.05 generally indicates the absence of remodeling, and the further the RI from 1, the more severe the extent of vascular remodeling [49,50,51,52,53]. For this indicator, the imaging depth should be deep enough to visualize the overall arterial structure, especially for the positive remodeling case. In addition, IVUS imaging should have the ability to clearly delineate the area of the media, which requires high contrast resolution. Many studies on arterial remodeling have been carried out, and the results show that positive remodeling is more dangerous than negative remodeling in that positive remodeling is closely associated with plaque rupture [52,53,54,55,56].

### 2.3. Thickness of Thin Fibrous Cap

**Figure 3:**
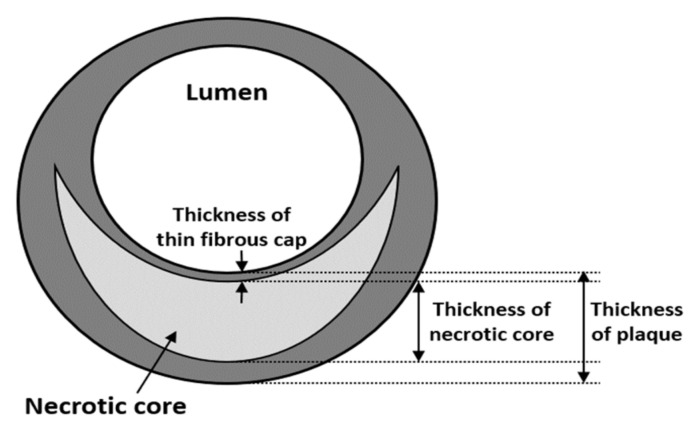
Thicknesses of the thin fibrous cap and necrotic core [67].

The thin fibrous cap literally denotes a fibrous cap with a thin thickness, as shown in Figure 3. When the plaque and inflammation are excessively accumulated, the fibrous cap becomes thin, making an increase in plaque vulnerability possible. Therefore, the thickness of the thin fibrous cap can be used as an indicator to predict plaque rupture. Virmani et al. [57] defined the thickness of a “thin” fibrous cap as less than 65 µm based on a pathological study by Burke et al. [58] that used a series of 41 ruptured plaques. Currently, this criterion is most commonly used to assess the stability of plaque [59,60,61,62,63,64,65,66]. The accurate measurement of the thin fibrous cap relies on the spatial resolution. For this, the center frequency of IVUS imaging should be higher than 70 MHz, which is challenging for current IVUS imaging systems. 

### 2.4. Thickness of Necrotic Core

Figure 3 shows the thicknesses of the necrotic core and plaque. The relationship between the thickness of the necrotic core and plaque vulnerability was studied by Ohayon et al. [67]. Using 24 in-vivo IVUS studies and a large set (n = 5500) of idealized plaque morphologies under the positive remodeling condition, the dominant morphological factors affecting plaque stability were assessed. A computational simulation was employed, and the threshold stress value that triggers plaque rupture was assumed to be 300 kPa based on previous studies [68,69]. The results showed that, when the RI is small, lesions with a relative core thickness (100 × core thickness ÷ plaque thickness measured at the location of the thin fibrous cap) of more than 50% were more prone to rupture. In other words, in the early stages of atherosclerosis, plaque vulnerability mainly relies on the thickness of the necrotic core rather than the area of the necrotic core or thickness of the fibrous cap. Similar studies have been carried out and support the results that the thickness of the necrotic core has an impact on plaque rupture [70,71,72,73]. For this clinical indicator, IVUS imaging should be able to distinguish the necrotic core from the plaque compositions, which requires accurate tissue characterization. This can be performed by detecting changes in the intensity and spectrum of the ultrasound reflected from each blood vessel layer. However, the identification of the necrotic core is currently limited in IVUS imaging due to its inaccurate border detection.

### 2.5. Vasa Vasorum Density

Vasa vasorum (VV) indicates a collection of microsized vessels with a diameter up to approximately 100–300 µm that forms to deliver nutrients and oxygen to vessel walls that have difficulty receiving sufficient nourishment from the lumen [74,75,76,77,78]. In the 1930s, some studies questioned the role of VV in the development of atherosclerosis, but they did not receive much attention at that time [79,80]. After a few decades, VV has been reported to be closely associated with the development of atherosclerosis by promoting inflammatory processes, resulting in an increase in plaque vulnerability. Therefore, at present, assessing the density of VV has also been considered as a diagnostic indicator [81,82,83,84,85,86,87,88]. Since VV is very small in diameter and difficult to separate from other tissues in IVUS B-mode images, contrast agents are commonly used to visualize it. 

### 2.6. Stress on Vessel Wall and Plaque

Because the blood flows inside the lumen, vessel walls are always stressed by the flow. It is not easy to assess stress on the vessel wall and plaque using only the IVUS image, but a computational analysis based on IVUS images can draw the stress values. 

Wall shear stress (WSS) indicates the force per lumen area and is calculated as [87,88]:

(2) $${\vec{\tau }}_{w}=\mu \left(\frac{∆\vec{V}}{∆r}\right)$$

where ${\vec{\tau }}_{w}$, μ, $\vec{V}$, and r are the WSS, absolute viscosity, velocity parallel to the wall, and radial distance from the wall, respectively. The WSS is closely related to the development of atherosclerosis. For example, in the early stage of atherosclerosis, low and oscillatory WSSs that have stress values less than 1 Pa lead to inflammation and dysfunction of ECs, whereas, in the late stage, a WSS value of more than 7 Pa indicates erosion, which can be used to diagnose atherosclerosis [89,90,91,92,93,94].

The main reason for plaque rupture is excessive mechanical stress on the fibrous cap. In other words, plaque ruptures when mechanical stress is applied above the threshold stress value of the fibrous cap; therefore, it is critical to assess the stress value on the plaque. Moreover, peak cap stress (PCS) is associated with the geometry of the plaque and vessel; therefore, PCS has also been used to determine morphological predictors [70,71,73,95]. In this regard, many studies have been carried out, and the threshold value of 300 kPa is frequently referred to by many studies [61,67,72,96,97]. IVUS elastography may be used for PCS measurement, but its clinical usefulness is still questionable. 

## 3. Stent Implantation with IVUS Image

IVUS image has also been used for the real-time guidance of percutaneous coronary intervention (PCI), i.e., monitoring pre- and post-stent surgery. During stent implantation, IVUS imaging helps to choose the optimal stent size based on cross-sectional IVUS images of the target blood vessels. Also, it can be used for minimizing the risk of stent thrombosis based on imaging information about the state of the stent in the blood vessel after the surgery, such as stent under-expansion or malapposition [98,99]. For efficient stent implantation, clear identification of stent struts apposed to the vessel wall is essential, which requires the high spatial and contrast resolution of IVUS imaging. High-frequency IVUS transducers are desirable for high spatial resolution. Also, multifrequency IVUS transducers can improve both spatial and contrast resolutions because tissue harmonic imaging and frequency compounding can be employed [100,101].

## 4. IVUS Transducers

### 4.1. Classification of IVUS Transducer

**Figure 4:**
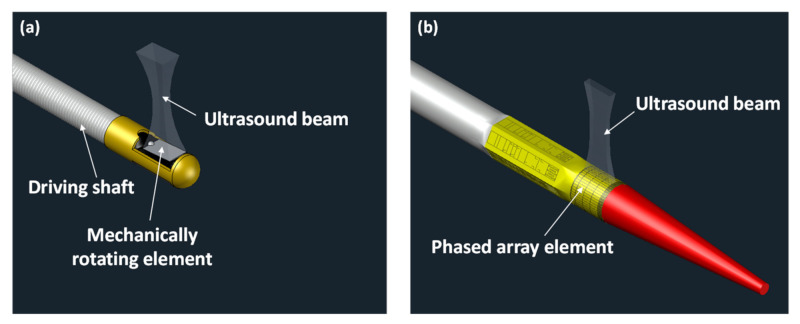
Schematic diagrams of the IVUS transducers: (**a**) mechanical IVUS transducer and (**b**) solid-state IVUS transducer.

Generally, IVUS transducers can be classified into two types, as shown in Figure 4: mechanical and solid-state IVUS transducers. The main difference between the two types of IVUS transducers is the method of image acquisition. Many advantages and disadvantages can be found when they are compared [102,103,104,105]. 

The mechanical IVUS transducer obtains the vascular cross-sectional image by 360° rotation with more than 1800 rpm, so it can form an image with only a single element. As a result, the mechanical IVUS transducer can be small (<1 mm in diameter) and thus minimize the size of an IVUS catheter (<2 mm in diameter) in which the transducer is rotated to generate and receive ultrasound [106]. In addition, the rigid tip of IVUS transducers is less than 2 mm in length [107]. These dimensions enable the catheter to pass easily through a stenosed vessel for the cross-sectional vascular image. However, because the image is acquired through mechanical rotation within the blood vessel, the image may be distorted, especially when it forms an image in a severely curved region. Non-uniform rotation may cause image artifacts that hamper accurate analysis of morphological features quantitatively. In addition, small bubbles, generated between the catheter sheath and the IVUS transducer due to the rotation, disrupt the transmission and reception of ultrasound, thus lowering image quality; saline flushing is often used to solve the problem [108,109]. The rotational scanning also requires a sophisticated design for electrical connection between a rotating IVUS transducer and a stationary imaging system to prevent twisting signal wires. For this, an electric brushed slip ring can be used to connect an IVUS transducer (i.e., a rotor) and a system (i.e., a stator) based on a sliding brush contact [107]. Since the rotor and the stator are in mechanical contact with each other in a slip ring, a sophisticated design is required to reduce friction and heat generation at the contact surfaces, especially when operating at a high frame rate. As an alternative, a rotary transformer (also called a wireless slip ring) can be used [110], which is a noncontact electromechanical interface. This method is based on the electromagnetic induction between the stator and the rotor separated by a narrow air gap. Since a rotary transformer has no physical contact, high friction and heat generation are no longer a problem. However, the implementation of this electromechanical interface that can minimize the loss of signal-to-noise ratio (SNR) over broadband is still challenging [110]. 

A solid-state IVUS transducer, which is also called a “multi-element phased array IVUS transducer,” builds the image by electrical control of each element. Therefore, it has good lateral resolution owing to the narrow lateral beamwidth, and there is no image distortion caused by rotational movement. However, the drawbacks of the solid-state IVUS transducer include a relatively large length of the rigid tip (e.g., 7 mm) [111], compared with the mechanical one because of integrated circuits inserted into the transducer and many signal wires. Note that their diameter is similar to that of mechanical IVUS catheters [106]. For the solid-state IVUS transducer, additionally, many conditions must be met, such as a complex system to control each element, as well as aspect ratio or pitch width, at a high fabrication cost. To this end, current commercial solid-state IVUS transducers have a center frequency of 20 MHz (Philips, Eagle Eye^®^, San Diego, CA, USA), thus resulting in poor axial resolution (<170 μm) [112].

### 4.2. Typical Structure of Mechanical IVUS Transducers

Similar to other ultrasound transducers, an acoustic stack of mechanical IVUS transducers consists of piezoelectric, matching, and backing layers. The piezoelectric layer generates and receives ultrasound, and its thickness determines the frequency of the transducer based on the following Equation (3) [113].

(3) $$f_{0}=\frac{nC_{p}}{2L}$$

where $C_{p}$ and L are the sound velocity and thickness of the piezoelectric layer, respectively, and n is an odd integer. When n = 1, it is called the “fundamental frequency,” which has the highest efficiency. 

The matching layer is designed to optimize the transmission of ultrasound to the medium, so its thickness and acoustic impedance are critical factors. Generally, the matching layer has a quarter-wavelength thickness for 100% transmission for a monochromatic plane wave, and its ideal acoustic impedance can be calculated as described in previous research [113,114].

(4) $$Z_{m}={\left(Z_{p}Z_{1}{}^{2}\right)}^{1/3}$$

where $Z_{m}$, $Z_{p}$, and $Z_{1}$ are the acoustic impedances of a single matching layer, piezoelectric layer, and medium, respectively. 

In the case of two matching layers, the ideal acoustic impedance of each layer is given as [113,114]:

(5) $$Z_{m1}={\left(Z_{p}{}^{4}Z_{1}{}^{3}\right)}^{1/7}$$

(6) $$Z_{m2}={\left(Z_{p}Z_{1}{}^{6}\right)}^{1/7}$$

where $Z_{m1}$ is the acoustic impedance of the first matching layer attached to a piezoelectric layer, and $Z_{m2}$ is the impedance of the second matching layer. Although multiple matching layers generally improve the performance of a transducer more than a single layer, optimal acoustic matching materials used for IVUS transducers are difficult to find because of their small size and unique usage environment. For two matching layers, silver-based conductive epoxy and parylene generally serve as the first and second matching materials, respectively [104,115,116]. 

The backing layer reduces the vibration of the piezoelectric layer and prevents the reflected signal from the rare face of the backing layer. Therefore, the acoustic impedance and the attenuation coefficient are important parameters. 

Generally, an IVUS transducer is mounted on a shaft and contained in an IVUS catheter that is 5F–6F in size to access the stenosed vessel readily, so the size of the IVUS transducer is restricted to less than 1.2 mm [106]. Under this condition, micro-coaxial cable is connected to the acoustic stack within the miniaturized IVUS housing; therefore, materials available for IVUS transducers are extremely limited. This implies that the electrical conductivity features should be further considered. 

**Figure 5:**
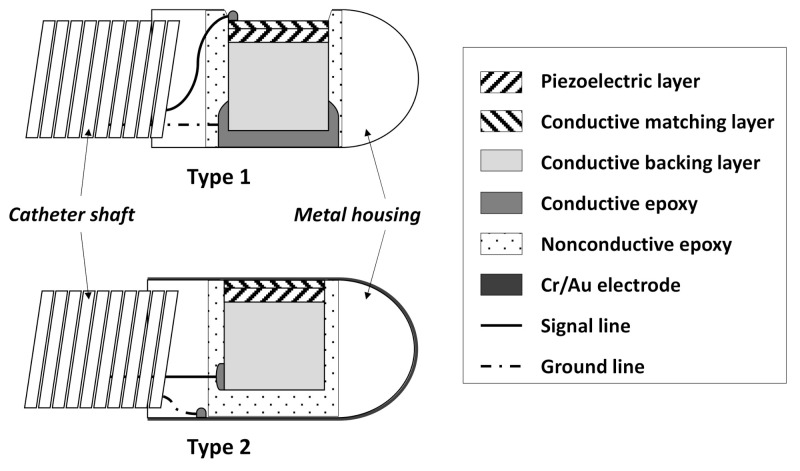
Structures of IVUS transducer depending on methods for signal line connection.

To connect the signal and ground lines to the acoustic stack, a coaxial cable was used. Based on the method for connecting the signal and ground lines, the structure is mainly divided into two parts, as shown in Figure 5. In type 1, the signal line is attached to the front face of the transducer aperture, and the ground line is connected to a conductive backing layer, a metal housing, and a shaft. Type 2 is a signal line attached to the backing layer, and the ground line is formed by the Cr/Au sputtering method covering the front face of the transducer aperture and the metal housing. Current commercial IVUS transducers employ a type 1 structure considering the mass-production process and fabrication cost, and type 2 structures are extensively exploited in the laboratories of universities [117]. 

### 4.3. Common Issues in Developing Mechanical IVUS Transducers

To improve the accuracy of diagnosis of atherosclerosis, IVUS transducers have been consistently developed to increase the spatial and contrast resolutions and the imaging depth. The spatial resolution is closely related to the center frequency of the IVUS transducer as follows [113,114].

(7) $$R_{axial}=\frac{c}{2BW_{-6dB}}$$

(8) $$R_{lateral}\approx \;\lambda f_{\#}$$

where $R_{axial}$ and $R_{lateral}$ are the axial and lateral resolutions, respectively, *c* is the sound velocity in the medium, *BW*_−6__*dB*_ is the −6-dB fractional bandwidth of the transducer, λ is the wavelength in the medium, and $f_{\#}$ is the f-number, which is the focal length divided by the aperture size. Therefore, for high spatial resolution, the center frequency and −6-dB fractional bandwidth should be increased. Unfortunately, attenuation depends on the frequency in biological tissues, and high-frequency (HF) IVUS transducers have suffered from shortened imaging depth, which causes some difficulties in providing overall morphological information of vessels that are used to construct arterial remodeling, to measure heavy plaque burden in the adventitia, or to choose the right type of stent [113,114,118,119]. Consequently, achieving both high spatial resolution and deep imaging depth is challenging. 

Limited material options for the IVUS transducer are an issue, with another one being IVUS catheter sheath. An IVUS transducer is placed inside a flexible translucent polymer sheath (e.g., polymethylepentene (TPX), polyurethane (PU), or polyether block amide (PEBAX)) filled with saline solution as an acoustic coupling medium, and sheaths have a low acoustic impedance (e.g., 1.5–1.9 MRayls) [120,121,122,123,124]. However, the effect of the sheath on image quality cannot be ignored. In other words, IVUS transducers should have sufficient sensitivity because an IVUS catheter sheath incurs a reduction in ultrasound energy by attenuation in the sheath and reflection at the surface of the sheath. It has been reported that conventional IVUS transducers are tilted at 0–8° to avoid sheath reflection [125], and the thickness of a catheter sheath through which ultrasound beam passes is less than 0.01 inch to minimize ultrasound attenuation [126,127,128]. Generally, IVUS catheter sheaths have 6–10 dB of roundtrip attenuation for 20–80 MHz [129], and Munding et al. [130] showed that the minimum insertion losses were increased by 7 and 12 dB at 30 and 80 MHz, respectively, when the transducers were parallel to the sheath. Thus, the higher the center frequency of the transducer, the more important it is to consider the sheath effect.

As a result, considering the above issues, the specifications of IVUS transducers (e.g., center frequency, shape and size of the aperture, and active and passive materials) should be selected carefully for sufficient sensitivity, resolution, and imaging depth for high-quality IVUS imaging. Here, the focus is on the study of mechanical IVUS transducers using piezoelectric ceramics or single crystals because commercial IVUS transducers are mainly composed of ceramics or single crystals and usually adopt mechanical IVUS transducers for high center frequencies. Although piezoelectric micromachined ultrasound transducers (pMUTs) and capacitive micromachined ultrasound transducers (cMUTs) can be candidates for IVUS imaging, however, they will not be discussed here.

### 4.4. Single-Frequency IVUS Transducers

Single-frequency IVUS transducers are typically composed of a single element with an operating frequency of higher than 20 MHz. Owing to the limited passive material options, there have been many efforts to develop optimal piezoelectric materials for IVUS transducers. Piezoelectric materials with a high electromechanical coefficient and high dielectric permittivity make possible broadband characteristics, high sensitivity, and good electric impedance matching under small aperture sizes. Piezoelectric materials, such as Pb(Zr, Ti)O_3_ (PZT)-based ceramics [131,132,133], Pb(Mg_1/3_Nb_2/3_)O_3_–PbTiO_3_ (PMN–PT) [115,131,134], and LiNbO_3_ [135,136,137,138], have been widely used for HF ultrasound transducers. However, conventional piezoelectric materials were unable to match the desired performances for IVUS transducers, and many researchers have developed synthesis methods for new piezoelectric materials. Li et al. [139] reported an 80-MHz IVUS transducer using a novel PMN–PT free-standing film that not only provides a high electromechanical coefficient (k_t_ = 0.55) and dielectric permittivity (ε_r_/ε_0_ = 4364) but also prevents the degradation of piezoelectric properties by a time-consuming lapping process. Yan et al. [140] suggested a 30-MHz IVUS transducer employing [Ba(Zr_0.2_Ti_0.8_)O_3_]_0.5_[(Ba_0.7_Ca_0.3_)TiO_3_]_0.5_ (BZT–50BCT) having superior piezoelectricity with a d33 value of approximately 600 pC/N. Zhang et al. [141] reported a 42-MHz miniaturized transducer using Pb(Ni_1/3_Nb_2/3_)O_3_–Pb(Zr_0.3_Ti_0.7_)O_3_ (PNN–PZT) with a high electromechanical coefficient (k_t_ = 0.60) and a high relative clamped dielectric permittivity (ε_S_/ε_0_ = 3409). PbIn_1/2_Nb_1/2_O_3_–PbMg_1/3_Nb_2/3_O_3_–PbTiO_3_ (PIN–PMN–PT) single crystal was used by Li et al. [142] because it can provide superior electrical and thermal stability by lead indium niobite (PIN) [143]. Using a customized piezoelectric material is a good option to improve the performance of IVUS transducers, but it is inevitably accompanied by a relatively complex synthetic process. The properties of the piezoelectric materials developed to improve their performance are summarized in Table 1. Despite the efforts to develop high-performance piezoelectric materials, the –6 dB fractional bandwidth of IVUS transducers is narrow, ranging from 30 to 50% [144]. This is mainly because only a few passive materials can be used for IVUS transducers [145], and thus it is difficult to optimally design a high-performance IVUS transducer with limited passive materials. As mentioned in Section 4.2, it should be noted that passive materials for acoustic matching layers are preferred to have low ultrasound attenuation at the high operating frequency and those for backing layers should be conductive to allow for easy connection between a piezoelectric material and a signal wire inside the small housing. 

Another way to improve the performance of the piezoelectric layer is to adopt a composite structure, in which piezoelectric ceramic and polymer are alternately arranged [146,147]. The composite structure offers a high electromechanical coefficient, thus providing high sensitivity and broad bandwidth characteristics by minimizing radial mode vibration. In addition, it provides good acoustic impedance matching to a medium like the human body (≈1.5 MRalys) because the polymer structure is distributed to reduce the overall acoustic impedance from >30 to 4–25 MRayls [113]. Finally, the composite structure is more flexible than the bulk structure, which facilitates geometrical deformation of aperture with minimal damage to the ceramic [148,149,150,151,152]. Because of these advantages, composite structures have been used in various IVUS transducers. In 2006, Yuan et al. [153] developed a 40-MHz PMN-PT-based 1–3 composite transducer using a deep reactive ion etching technique. Two years later [154], the same fabrication technique was used to develop a 60-MHz IVUS transducer with a high electromechanical coefficient (k_t_ = 0.72) when it had a volume fraction ratio of approximately 40%. In 2014, Li et al. [142] applied a 1–3 composite structure to a PIN-PMN-PT single crystal for a 41-MHz IVUS transducer. One of the inconveniences of employing composite structures is that it is difficult to secure the conditions of the composite structure, such as kerf and ceramic widths, to avoid lateral mode vibration as the frequency increases. For example, in a 1-3 composite structure, to avoid lateral mode vibration, the kerf and ceramic width should be less than v_S_/(2f) and v_L_/(2f), respectively, where v_S_, v_L_, and f are the shear wave velocity of the polymer filler, lateral wave velocity of the piezoelectric element, and thickness resonant frequency of the composites [155], respectively. For example, for an 1–3 piezo-composite structure for a center frequency of 60 MHz, a kerf width should be less than 10 μm. However, the minimal blade width of a commercial dicing saw is larger than 10 μm (e.g., 15~20 μm). As a result, the mechanical dicing technique cannot be used in HF transducers (>60 MHz) unless complicated fabricated methods such as the interdigital pair bonding for 1–3 composite is used [156], so an etching technique is required.

**Figure 6:**
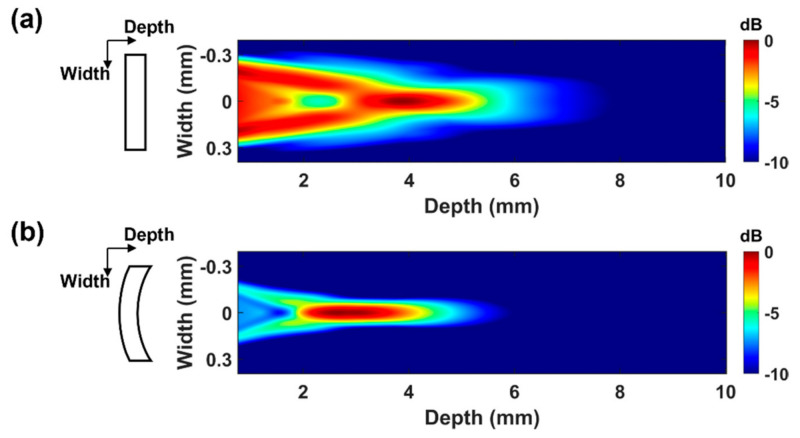
One-way radiation patterns of 80-MHz IVUS transducer with 0.6-mm aperture diameter according to aperture shape. (**a**) IVUS transducer with flat aperture and its one-way radiation pattern, and (**b**) IVUS transducer with spherically press-focused aperture and its one-way radiation pattern.

Using geometrical ultrasound beam focusing is one way to improve IVUS image quality. For this, either an acoustic lens or a spherical transducer aperture is commonly used for single-element transducers. For IVUS transducers, however, acoustic lenses are not suitable because HF ultrasound is attenuated more in lens materials and it is difficult to attach a lens to very small transducers. Geometrical focusing can be applied to HF transducers by shaping aperture, which can be conducted using the press-focusing method; a spherically curved aperture can be formed by pressing the aperture with a steel sphere [157,158]. By focusing the ultrasound beam, a geometrical focused IVUS transducer can achieve high sensitivity and the best lateral resolution, resulting in superb contrast resolution in the region of interest. Another reason for using geometrical beam focusing in the HF IVUS transducer is to locate the ultrasound beam focus at the proper depth for the detection of a thin fibrous cap. Since the fibrous cap is located under the endothelium of the vessel, ultrasound focus should be situated 1~2 mm from the lumen wall. With a given aperture size, the natural focal depth defined as the boundary between the Fresnel zone and the Fraunhofer zone is proportional to the center frequency [113,114], i.e.,

(9) $$Z_{0}=\frac{D^{2}}{4\lambda }=\frac{D^{2}}{4}\cdot \frac{f}{c}$$

where $Z_{0}$, D, λ, c, and f are the natural focal depth, aperture diameter, wavelength, sound speed in the medium, and frequency, respectively. Note that optimal ultrasound imaging can be performed in the Fraunhofer zone if an unfocused transducer is used. Figure 6 shows the one-way radiation patterns obtained by finite-element-analysis-based simulation (Onscale, Cupertino, CA, USA). A disk-shaped aperture with a 0.6-mm diameter was used, and the center frequency was set to 80 MHz. Based on Equation (9), the natural focus of the flat aperture transducer is formed at 4.8 mm deeper than where the fibrous cap is located (see Figure 6a). Therefore, beam focusing is not located at the desired position for the detection of a fibrous cap. On the other hand, the geometrically focused transducer can form a focal region within the near field (also called the Fresnel zone). As a result, the best lateral resolution can be obtained around the position of the fibrous cap. Note that spherical geometric focusing can improve the lateral resolution, even though the geometrical focal point is set to around the natural focal depth [116]; the focal region of the radiation pattern created by a spherically shaped transducer with a radius curvature of 4.8 mm (i.e., the natural focal depth) was around 3 mm, as shown in Figure 6b.

Yoon et al. [104] and Lee and Chang [159] suggested angled press-focused IVUS transducers with a square aperture to improve the resolution and accessibility to the target region. In addition, a 50-MHz oblong press-focused IVUS transducer was proposed by Lee et al. [116] to improve the contrast resolution by reducing the slice thickness (i.e., beam thickness in the elevation direction). In the same year, Jian et al. [157] reported a 50-MHz press-focused IVUS transducer with a 1–3 composite structure for both improved performance and prevention of microcracks during a press-focusing process. 

One of the disadvantages of geometrical focusing is that the beam width becomes more variable in-depth, resulting in a shortening of the depth of focus. In other words, the beamwidths in the near and far-fields are much greater than in the focal region, resulting in a nonuniform contrast resolution and signal-to-noise ratio. Therefore, the focal point should be determined carefully, considering the imaging depth. Table 2 presents a summary of the characteristics of single-frequency IVUS transducers.

### 4.5. Multifrequency IVUS Transducer

Frequency is the dominant factor affecting ultrasound images. Low-frequency (LF) IVUS images have high imaging depths at the expense of low spatial resolution, whereas HF images have the opposite. To achieve both advantages, multifrequency IVUS transducers have been developed. Multifrequency IVUS transducers make it possible to use various imaging techniques, such as harmonic imaging, acoustic radiation force impulse (ARFI) imaging, and superimposed multifrequency imaging. 

Typically, multifrequency IVUS transducers are composed of more than two piezoelectric layers. However, Vos et al. [160,161], and Frijlink et al. [162] proposed a dual-frequency IVUS transducer for harmonic imaging by using a special layer for the generation of a multifrequency spectrum. They used a “mismatching layer” or “tuning layer” to realize dual-frequency characteristics at 20 and 40 MHz, which is done to improve transmission efficiency during harmonic imaging. In this case, the layers for the dual-frequency spectrum cause attenuation by themselves, and there is a concern that sensitivity is degraded. In addition, most researchers have developed multifrequency transducers using multiple piezoelectric layers. According to the arrangement of the piezoelectric layers, they can be largely divided into stacked and enumerated types. 

For a given size of each element, the stacked type can minimize the overall aperture size of IVUS transducers, compared to the case where the elements are placed side by side. Usually, the overall aperture size is determined by the piezoelectric layer for LF, considering the electrical impedance of the transducer. Jiang et al. [163,164,165,166] explored a layered multifrequency IVUS transducer, which has a piezoelectric layer for LF underneath the layer for HF. Usually, they used the LF element for transmission and the HF element for the reception to accomplish contrast-enhanced superharmonic imaging [136,138,139]. For acoustic radiation force impulse (ARFI) imaging, additionally, the LF element was used for the generation of the pushing beam to induce displacement of the target, whereas the HF element was subsequently used for the transmission and reception of the tracking beam to measure the displacement [164]. In this configuration, it is necessary to insert an isolation layer between the HF and the LF elements to prevent signal ringing and thus image resolution degradation. This is so because the echo signal can be delivered to the LF element through the HF element during echo signal reception, thus causing the LF element to vibrate and affect the HF element vibration [167].

Sung and Jeong [168] reported a multifrequency IVUS transducer using the polarization inversion layer technique (PIT) for harmonic imaging. The PIT also provides a layered structure, but the difference from the above studies is that the two piezoelectric layers have opposite poling directions and are activated simultaneously, resulting in multiple resonances at the fundamental frequency. This is determined by the total thickness of the piezoelectric layers and their harmonic frequencies. Because the PIT utilizes the interaction between two piezoelectric layers, there is no need for an isolation layer. However, because of this mechanism, the sensitivity of the PIT-applied transducer has a median level between transducers with a single piezoelectric layer. In other words, when the PIT-applied transducer is activated by its harmonic frequency, it has lower sensitivity than a transducer with a single piezoelectric layer with the same frequency; however, when it is driven by its fundamental frequency, it has higher sensitivity than the conventional transducer [169]. 

The other configuration of the stacked-type multifrequency IVUS transducer is a back-to-back structure in which the HF and LF piezoelectric layers share the backing layer [130,170,171]. With this structure, the transducer can obtain two cross-sectional images with different frequencies and visualize the overlaid images in real-time by simply rotating either of them 180°. However, this structure also must be considered an undesirable interaction between the layers. 

In the enumerated type, elements with different resonances are usually arranged along the axis of the IVUS catheter sheath to avoid an increase in catheter diameter. Yoon et al. [172] proposed a dual-element IVUS transducer with separately press-focused elements for superimposed multifrequency IVUS images, and Shih et al. [173] presented a dual-element IVUS transducer for IVUS elastography. Thereafter, Lee et al. [100,145,174] developed an IVUS transducer composed of two or three elements sharing the same focal point for tissue harmonic imaging, frequency compounding imaging, and superimposed multifrequency imaging. Owing to the confocal structure, the proposed transducer could achieve harmonic signals more effectively than a multielement IVUS transducer with elements carrying their own image axis. With these configurations, considering the crosstalk effect between the elements and the increase in the rigid region at the catheter tip, the distance between the elements should be considered carefully [145].

Electrical impedance matching between an IVUS transducer and an imaging system can improve signal bandwidth and SNR [175]. Since the multifrequency IVUS transducers commonly consist of two elements with different frequencies, two different matching circuits are necessary. Especially, many efforts should be made to design an electrical impedance matching circuit for a HF element. This is so because the higher the operating frequency, the lower the electrical impedance (<20 Ω), thus lowering the efficiency of electrical power delivery. To overcome this issue, a cable-shared dual-frequency catheter was developed, in which a single signal wire was used to connect both HF and LF elements to an imaging system [176]. The signal connection in the developed catheter is equal to a parallel connection of two signal wires because the housing serves as a ground. As the parallel connection leads to a reduction in electrical impedance [177], series connection is preferred if both elements have an electrical impedance lower than a system impedance (typically 50 Ω). The developed multifrequency IVUS transducers are listed in Table 3. 

## 5. Summary and Outlook

The purpose of this review was to introduce the developed mechanical IVUS transducers with design considerations as well as the overall background related to diagnosis with the IVUS image, including the history of IVUS transducer, development of atherosclerosis, and diagnostic indicators of the disease.

IVUS transducers were developed in the middle of the 1900s and have been improved to detect atherosclerosis, especially in vulnerable plaques. Diagnosis of vulnerable plaque in the early stage is critical because more than 70% of acute coronary events, such as acute myocardial infarction or acute stroke, are caused by rupture of vulnerable plaques [23,24]. For this, various diagnostic indicators have been studied, and most of them have been derived from morphological features, even though some of them have pathological features, such as stress or stiffness values at the fibrous cap, with the aid of computer analysis. 

To detect the aforementioned diagnostic indicators such as plaque burden, remodeling index (RI), the thickness of fibrous cap and necrotic core, and vasa vasorum (VV) density, an IVUS image with advanced resolution and sensitivity obtained by an IVUS transducer is required; therefore, the performance of the IVUS transducer is most important. Typically, IVUS transducers are classified into mechanical IVUS transducers rotating 360° within the vessel and solid-state IVUS transducers that take the vessel image without rotation. The solid-state IVUS transducer comprises multiple elements controlling each element by an electrical signal, so it makes it possible to achieve good lateral resolution without any image distortion caused by rotation. However, the demands of a complex IVUS system, high fabrication cost to control each element, and inevitable increase in the rigid tip length of an IVUS catheter have impeded vigorous study of solid-state IVUS transducers. However, mechanical IVUS transducers can maintain a minimal rigid area at the catheter tip, low fabrication cost, and simple fabrication process compared with solid-state transducers; therefore, studies on mechanical IVUS transducers have been widely conducted. 

A mechanical IVUS transducer can be categorized as a single-frequency transducer and a multifrequency transducer. The single-frequency transducer has a single piezoelectric layer capable of minimizing the rigid tip length and has an easy fabrication process in comparison with the multielement type. To improve the performance of single-frequency IVUS transducers, researchers have developed piezoelectric materials with a high electromechanical coefficient and high dielectric permittivity and have attempted to adopt a piezo-composite structure. In addition, ultrasound beam focusing using a geometrically curved piezoelectric layer can provide improved spatial/contrast resolution and enhanced sensitivity at the focal area. 

With single-frequency transducers, there is a limit to expanding the frequency bandwidth, which makes possible the application of various imaging processes, such as harmonic imaging, frequency compounding imaging, superimposed multifrequency imaging, or ARFI imaging, to IVUS transducers. Depending on the configuration of the IVUS transducer, it can be categorized as a stacked type or an enumerated type. The aperture size of the stacked type usually depends on the element size with a low center frequency. In the enumerated type, elements are arranged along the catheter axis. There is a high possibility of increasing the aperture size or rigid part of the catheter sheath when a multifrequency structure is used, but there is no restriction on choosing the frequency combination if two different piezoelectric layers are used. Even though the total aperture size of the transducer or rigid part of the catheter sheath is somewhat increased, the interest in and demand for multifrequency IVUS transducers will be prolonged for some time.

Future developments will be for high-performance IVUS transducers with a small aperture size, high sensitivity, high center frequency, and broad bandwidth for advanced IVUS images capable of providing more accurate diagnostic information with minimized patient inconvenience during the surgery. Therefore, it is natural that the next generation of mechanical IVUS transducers should consist of more than one element considering the advantages of multifrequency IVUS transducers It is expected that consistent efforts to maintain small aperture sizes will be made. Among the various techniques for this, microelectromechanical system (MEMS) technology is one of the most promising for realizing a small-aperture IVUS transducer. Typically, the MEMS technique has been applied to solid-state IVUS transducers [178,179,180,181,182,183], but it could also be a good candidate for addressing size issues for IVUS transducers with multiple elements. 

However, there are still limitations in accurately diagnosing atherosclerosis using IVUS imaging alone. Thus, more than one imaging modality has been integrated into the IVUS images. For example, IVUS/intravascular optical coherence tomography (IV-OCT) [184,185,186,187,188,189] and IVUS/near-infrared spectroscopy (NIRS) [190,191,192,193,194] are currently available for practical use, and IVUS images with near-infrared fluorescence (NIRF) [195,196,197,198,199], fluorescence lifetime imaging (FLIm) [200,201,202,203,204], and intravascular photoacoustic (IVPA) [123,205,206,207,208] have been investigated at the laboratory level. These combinations can complement the drawbacks of each imaging modality while providing more clinical information. For example, IV-OCT has superior spatial resolution but has a low imaging depth, and the other imaging modalities can provide functional information with a lack of structural information. Therefore, research on hybrid intravascular imaging techniques has become more prominent. Quantitative performance comparison of atherosclerosis imaging modalities including angiography [209] is summarized in Table 4. 

In addition: the IVUS image can also be improved by optimizing the properties and specifications of the catheter sheath, which is the window through which the ultrasound beam or other imaging source passes, or by minimizing image distortion caused by rotation of the transducer or the pulsation. Thus, it may be a long journey to increase accuracy in the diagnosis of atherosclerosis, especially for high-risk plaques, but the goal will be achieved through continuing research.

## Figures and Tables

**Table 1:** Properties of various piezoelectric materials used in intravascular ultrasound (IVUS) transducers.

Piezoelectric Materials	k_t_	d_33_ (pC/N)	ε_S_/ε_0_	c (m/s)	ρ (kg/m^3^)
PZT–5H [131]	0.51	593	1470	4580	7500
PMN–33%PT single crystal [115]	0.58	1430	797	4608	8000
LiNbO_3_ single crystal [137]	0.49	-	39	7340	4640
PMN–PT free-standing film [139]	0.55	-	-	-	7760
BZT–50BCT [140]	0.41	597	2817	5133	5200
PNN–PZT-based ceramic [141]	0.60	760	3409	3880	7781
PIN–PMN–PT single crystal [143]	0.59	2742	659	4571	8198

Note: Here, k_t_, d_33_, ε_S_/ε_0_, c, and ρ are the electromechanical coupling coefficient in thickness mode, the piezoelectric coefficient, the clamped dielectric constant, the longitudinal wave velocity, and density in the order.

**Table 2:** Characteristics of single-frequency mechanical IVUS transducers.

Categories	Center Frequency	−6-dB Bandwidth	$\mathrm{Acoustic}\;\mathrm{Impedance}\;\mathrm{of}\;Z_{p}$	Volume Fraction	k_t_	Focal Length	Aperture Size	Axial Resolution	Lateral Resolution
Using high-performance piezoelectric material	82 MHz [139]	65%	-	-	55	-	0.4 × 0.4 mm^2^	35 μm	176 μm
	30.5 MHz [140]	53%	26.7 MRayls	-	41	-	0.8 × 0.8 mm^2^	-	-
	42 MHz [141]	79%	30.2 MRayls	-	60	-	0.33 × 0.33 mm^2^	36 μm	141 μm
Using 1-3 composite structure	41 MHz [142]	77%	17.8~21.5 MRayl	65 ± 5%	71 ± 4	-	-	-	-
	60 MHz [154]	77%	<20 MRayls	~40%	70	-	< 0.6 ∅ mm	-	-
	41 MHz [153]	86%	20~22	70~80%	75~78%	-	0.5 × 0.4 mm^2^	43 μm	226 μm
Using geometrical focusing technique	47 MHz [104]	72%	-	-	-	2.5 mm	0.57 × 0.57 mm^2^	25 μm	120 μm
	52 MHz [116]	41%	36.7 MRayls	-	-	3 mm	0.5 × 1.0 mm^2^	-	180 μm
	37 MHz [159]	62%	36.7 MRayls	-	-	3 mm	0.5 × 0.5 mm^2^	58 μm	211 μm
	52 MHz [157]	107%	-	36%	50%	3 mm	0.6 × 0.6 mm^2^	80 μm	100 μm

**Table 3:** Characteristics of multifrequency mechanical IVUS transducers.

Categories	Type	Frequency Combination (LF/HF)	Each Element Aperture Size	Total Aperture Size	Focal Length	Application
Stacked structure	Layered/unfocused [160,161]	22 MHz/40 MHz	-	0.75 × 1.0 mm^2^	-	Tissue harmonic imaging
	Layered/unfocused [168]	17 MHz/34 MHz	-	0.60 × 1.0 mm^2^	-	Tissue harmonic imaging
	Layered /unfocused [163]	6.5 MHz/30 MHz	0.60 × 3.00 mm^2^ /0.60 × 0.50 mm^2^	0.60 × 3.00 mm^2^	-	Super harmonic contrast imaging
	Layered/unfocused [164]	5 MHz/40 MHz	0.60 × 3.00 mm^2^ /0.60 × 0.60 mm^2^	0.60 × 3.00 mm^2^	-	ARFI imaging
	Layered/unfocused [165]	6.5 MHz/30 MHz, 5.0 MHz/30 MHz, 5.0 (1–3 composite) MHz/30 MHz	0.60 × 3.00 mm^2^ /- mm^2^	0.60 × 3.00 mm^2^	-	Super harmonic contrast imaging
	Layered/unfocused [166]	2.25 MHz/32 MHz	0.37 × 5.00 mm^2^ /0.37 × 0.50 mm^2^	0.37 × 5.00 mm^2^	-	Super harmonic contrast imaging
	Back-to-back/unfocused [170]	35 MHz/90 MHz, 35 MHz/120 MHz, 35 MHz/150 MHz	0.50 × 0.50 mm^2^ /0.50 × 0.50 mm^2^	0.50 × 0.50 mm^2^	-	Superimposed multi-frequency imaging
	Back-to-back/unfocused [130,171]	34 MHz/79 MHz	0.50 × 0.50 mm^2^ /0.27 × 0.27 mm^2^	0.50 × 0.50 mm^2^	-	Superimposed multi-frequency imaging
Enumerated structure	Focused (each) [172]	48 MHz/152 MHz	0.57 × 0.57 mm^2^ /0.57 × 0.57 mm^2^	0.50 × 2.07 mm^2^	2.5 mm	Superimposed multi-frequency imaging
	Unfocused [173]	8.5 MHz/31 MHz	2.00 × 3.00 mm^2^ /1.00 × 1.00 mm^2^	2.00 × 4.50 mm^2^	-	ARFI imaging
	Focused (together) [145]	35 MHz/70 MHz	0.50 × 0.50 mm^2^ /0.50 × 0.50 mm^2^	0.50 × 1.70 mm^2^	3 mm	Tissue harmonic imaging
	Focused (together) [174]	35 MHz/105 MHz	0.50 × 0.50 mm^2^ /0.50 × 0.50 mm^2^	0.50 × 1.10 mm^2^	2.5 mm	Tissue harmonic imaging
	Focused (together) [100]	35 MHz/70 MHz	0.50 × 0.50 mm^2^ /0.50 × 0.50 mm^2^	0.50 × 1.10 mm^2^	3 mm	Tissue harmonic imaging & Frequency compounded imaging

**Table 4:** Performance comparison of atherosclerosis imaging modalities.

	Angiography [209]	IVUS [170]	IV-OCT [189]	IV-NIRS [205]	IV-NIRF [198,199]	IV-FLIm [203,204]	IVPA [205]
Source	X-ray	Ultrasound	NIR light	NIR light	NIR light (Transmission) + Fluorescence (Reception)	NIR light (Transmitssion) + Fluorescence lifetime (Reception)	NIR light (Transmission) + Ultrasound (Reception)
Image plane	Projected side view	Cross-sectional view	Cross-sectional view	Cross-sectional view	Cross-sectional view	Cross-sectional view	Cross-sectional view
Imaging type	Morphological image	Morphological image	Morphological image	Molecular image	Molecular image	Molecular image	Molecular image
Imaging depth	N/A	<10 mm	<1~2 mm	Unknown	≈2~5 mm	≈0.2 mm	<5 mm
Axial resolution	N/A	<200 μm	≈10~20 μm	N/A	N/A	N/A	<100 μm
Lateral resolution	≈200 μm	<400 μm	≈20~90 μm	1000 μm	≈100 μm	≈100 μm	<500 μm

## Data Availability

Not applicable.
